# Emergence of Bluetongue Virus Serotype 3, the Netherlands, September 2023

**DOI:** 10.3201/eid3008.231331

**Published:** 2024-08

**Authors:** Melle Holwerda, Inge M.G.A. Santman-Berends, Frank Harders, Marc Engelsma, Rianka P.M. Vloet, Eveline Dijkstra, Rene G.P. van Gennip, Maria H. Mars, Marcel Spierenburg, Lotte Roos, René van den Brom, Piet A. van Rijn

**Affiliations:** Wageningen Bioveterinary Research, Lelystad, the Netherlands (M. Holwerda, F. Harders, M. Engelsma, R.P.M. Vloet, R.G.P. van Gennip, P.A. van Rijn);; Royal GD, Deventer, the Netherlands (I.M.G.A. Santman-Berends, E. Dijkstra, M.H. Mars, L. Roos, R. van den Brom);; Netherlands Food and Consumer Product Safety Authority, Utrecht, the Netherlands (M. Spierenburg);; North-West University, Potchefstroom, South Africa (P.A. van Rijn)

**Keywords:** bluetongue virus, bluetongue, sheep, cattle, serotyping, genotyping, vector-borne infections, viruses, zoonoses, the Netherlands

## Abstract

Since 1998, notifiable bluetongue virus (BTV) serotypes 1–4, 6, 8, 9, 11, and 16 have been reported in Europe. In August 2006, a bluetongue (BT) outbreak caused by BTV serotype 8 began in northwestern Europe. The Netherlands was declared BT-free in February 2012, and annual monitoring continued. On September 3, 2023, typical BT clinical manifestations in sheep were notified to the Netherlands Food and Product Safety Consumer Authority. On September 6, we confirmed BTV infection through laboratory diagnosis; notifications of clinical signs in cattle were also reported. We determined the virus was serotype 3 by whole-genome sequencing. Retrospective analysis did not reveal BTV circulation earlier than September. The virus source and introduction route into the Netherlands remains unknown. Continuous monitoring and molecular diagnostic testing of livestock will be needed to determine virus spread, and new prevention strategies will be required to prevent BTV circulation within the Netherlands and Europe.

Bluetongue virus (BTV) is an arthropodborne virus that can cause clinical disease and death in ruminants. All ruminants are susceptible to infection by BTV; infections in New World camelids have also been described (*1*–*3*). Other species, including humans, are not susceptible to infection, indicating that bluetongue (BT) is not a zoonosis.

BTV is transmitted by *Culicoides* spp. biting midges and has been historically present only between latitudes 35°S and 50°N (*4*,*5*). The BTV serogroup consists of >30 serotypes, of which serotypes 1–24 are notifiable to the World Organisation for Animal Health (WOAH). Since 1998, notifiable BTV serotypes 1–4, 6, 8, 9, 11, and 16 and nonnotifiable BTV serotypes 25 and 27 have been found in Europe and the Mediterranean Basin (*6*). In 2006, bluetongue virus serotype 8 (BTV-8) emerged in northwestern Europe, and the Netherlands was the first country where the virus infection was detected (*7*,*8*). After a major BT outbreak caused by BTV-8 in Europe during 2006–2007, an emergency BTV-8 vaccine became available in 2008 (*9*,*10*). Many owners of cattle herds and small ruminant flocks participated in the voluntary vaccination program conducted by the government of the Netherlands (*11*), resulting in a dramatic decline in the number of BTV clinical notifications to the Netherlands Food and Consumer Product Safety Authority (NVWA) in 2008. At the end of 2008, BTV antibodies were found in >80% of the susceptible host populations tested because of either natural infection or vaccination. No new infections were observed after 2009, and, after 3 years of screening for possible BTV circulation, the Netherlands regained its official BT-free status in February 2012. This BT disease–free status has been monitored annually according to European Union (EU) regulation 1108/2008/EC and has been confirmed without interruption up to December 2022. However, because of the risk of introducing BTV-8 from neighboring countries, vaccination has been authorized and, therefore, some farmers still vaccinate their animals for BTV-8.

On September 3, 2023, clinical signs in sheep indicative of BT were notified to authorities in the Netherlands simultaneously by 2 veterinary practices located within the middle of the country. We describe the questions raised and actions taken during the first 3 weeks after the initial notified clinical case was confirmed as a BTV infection by laboratory diagnostic tests.

## Methods

### Sheep and Cattle Populations in the Netherlands and Clinical Examination

In 2022, the Netherlands had ≈1,080,631 sheep and ≈1,596,894 dairy cattle >2 years of age, distributed among ≈31,000 sheep farms and 14,000 cattle herds (*12*,*13*). Farms that notified authorities of possible BT were visited by a veterinary team, specializing in small ruminants, who reviewed reported clinical symptoms and collected samples for BT diagnosis. In addition, several farms already confirmed as BTV-positive were visited by Royal GD (formerly the Gezondheidsdienst voor Dieren) personnel, who clinically examined the sheep and cattle on those farms and described clinical signs.

### Retrospective Study

We investigated whether the initial BT outbreak started in the area of the 4 BTV serotype 3 (BTV-3)–confirmed sheep farms in central Netherlands or whether the outbreak began earlier than September. We screened bulk tank cow milk samples submitted during August 2023 for routine testing from all over the Netherlands to determine if BTV antibodies were present. Royal GD coordinates a national monitoring program for which ≈12,000 (90%) dairy cattle farmers in the Netherlands submit monthly bulk tank milk samples. A total of 1,000 submitted milk samples were eligible for inclusion in the BTV screening.

We used identification and registration data from the Rijksdienst Voor Ondernemend Nederland (https://www.rvo.nl) to enable selection of dairy herd farms that did not purchase any cattle during the vector-active season (beginning in April 2023 until the start of the outbreak in September 2023) and only housed animals bred in the Netherlands. After applying the selection criteria, we were able to use bulk milk samples from 7,900 dairy herds for the screening. The Netherlands is divided into 20 compartments as proposed in the EU Commission Decision 2005/393/EC. We randomly selected 1,000 bulk milk samples to include all 20 compartments (Appendix Figure 1), resulting in ≈50 sampled herds per compartment and enabling a 14% prevalence estimate with 95% confidence. On September 11, we presented the first preliminary results to the government of the Netherlands. On September 13, additional data on vaccination purchases registered in the MediRund database (https://www.medirund.nl) during 2019 through September 2023 became available, and we combined those data with the results from the bulk milk screening.

### BTV Genome Detection by PCR

We performed BTV-specific real-time reverse transcription PCR (RT-PCR) as previously described (*14*). In brief, we extracted virus RNA from 200 µL of EDTA blood by using the Magnapure 96 robotic machine (Roche, https://www.roche.com) and MagnaPure 96 DNA and Viral NA Small Volume Kit (Roche). For RT-PCR, we combined 5 μL eluted RNA and 15 μL LightCycler RNA Master HybProbe kit (Roche) reagent containing enzymes and BTV-specific primers and probe and loaded each reaction mixture per well into a 96-well plate. We amplified the resulting cDNA by using a LightCycler 480 II instrument (Roche) and LightCycler integrated software version 1.5.1, without the external predenaturation step (*14*).

### Serologic Analysis using Competitive ELISA 

We performed a competitive ELISA by using an ID Screen Bluetongue Competition ELISA kit (Innovative Diagnostics, https://www.innovative-diagnostics.com) according to the manufacturer’s protocol. This ELISA has a sensitivity and specificity of 100% for BTV-specific antibodies but cannot detect antibodies generated against the genetically related epizootic hemorrhagic disease virus. We measured optical density at 450 nm by using a Multiskan FC instrument (Thermo Fisher Scientific, https://www.thermofisher.com) and MikroWin software version 5.09 (Labsis, https://labsis.de) and calculated the percentage inhibition by using the positive and negative controls supplied with the ELISA kit.

### Whole-Genome Sequencing

We extracted RNA from EDTA blood and amplified BTV genome segments by using a sequence-independent single-primer amplification approach. We performed first-strand cDNA synthesis by combining 5 µL RNA and Superscript III (Thermo Fisher Scientific) according to the manufacturer’s protocol and 2 μmol/L of the oligonucleotide 5′-GTTTCCCAGTCACGATA(N9)-3′. We incubated the mixture for 3 minutes at 95°C to denature double-stranded virus RNA, then cooled on ice. We added the remaining reagents and incubated the reaction at 25°C for 5 minutes, 42°C for 50 minutes, and 70°C for 15 minutes, and then stored at 4°C. We performed second-strand synthesis by using Sequenase (Thermo Fisher Scientific) according to the manufacturer’s protocol. We amplified the products by using Q5 high-fidelity DNA polymerase (New England Biolabs, https://www.neb.com) according to the manufacturer’s guidelines, 2 µmol/L of oligonucleotide 5′-GTTTCCCAGTCACGATA-3′, and the following cycle conditions: 94°C for 4 minutes; 68°C for 5 minutes; 35 cycles of 94°C for 30 seconds, 50°C for 1 minute, and 68°C for 3 minutes; then 68°C for 5 minutes and cooling at 10°C. To enhance the number of virus sequence reads, we performed a size selection of >200 bp by using SPRIselect beads (Beckman Coulter Life Sciences, https://www.beckman.com) at a ratio of 100 µL sample to 80 μL beads. We barcoded ≈150 ng cDNA from each sample by using the Native Barcoding Kit 96 V14 (Oxford Nanopore Technologies, https://www.nanoporetech.com) according to the manufacturer’s protocol and sequenced the cDNA by using a PromethION Flow Cell, R10 M version (Oxford Nanopore Technologies). To align the reads, we applied Minimap 2 version 2.26 (*15*) against a custom BTV reference database to construct a draft genome by using reference-based mapping. We deposited the sequences into GenBank on September 26, 2023 (accession nos. OR603992–4001).

We conducted phylogenetic analysis separately for each genome segment sequence by using BLAST (https://blast.ncbi.nlm.nih.gov) and the alignment results of the top 15 sequences used for the analysis. In addition, we added genome segment (Seg)-2 reference strains and a selected number of closely related BTV-3 strains to the phylogenetic analysis (*16*). We aligned the sequences by using MAFFT version 7.475 (*17*) and reconstructed the phylogeny by using maximum-likelihood analysis in IQ-TREE version 2.0.3 (*18*) and 1,000 ultrafast bootstrap replicates (*19*). We visualized the tree by using the ggtree R package (*20*).

### Antibody Detection in Bulk Tank Milk by Indirect ELISA

For the retrospective analysis of BTV antibodies in bulk milk, we used the ID Screen Bluetongue Milk Indirect ELISA (Innovative Diagnostics) according to the manufacturer’s protocol; this ELISA uses the recombinant VP7 protein as the antigen and was validated in the Netherlands in 2007 (*21*). To study herd prevalence of BTV-3 infections in 2023, we used the cutoff values described in the ELISA manual: sample/positive control (S/P) values of <30% were considered negative, S/P values of >30% to <40% were considered potential positives, and S/P values >40% were considered positive. Using those cutoff values, the test had a sensitivity of ≈95% and specificity of 100%.

### Geographic Distribution of BTV Clinical Cases

We graphically displayed sheep and cattle densities in thematic maps of the Netherlands according to 2-digit postal codes. We used BTV-confirmed clinical notifications of sheep and cattle cases until September 29, 2023. We generated all maps by using Stata version 17 (StataCorp LLC., https://www.stata.com).

## Results

### Timeline of Outbreak 

On September 3 and 4, 2023, NVWA was notified of clinical signs that were indicative of BTV infections at 5 sheep farms in the central region of the Netherlands near the Loosdrechtse Plassen. Flocks were visited by a team of veterinary specialists, and serum and EDTA blood samples were collected from the sheep and sent to the Netherlands National Veterinary Reference Laboratory for BTV at Wageningen Bioveterinary Research (WBVR).

**Figure 1:**
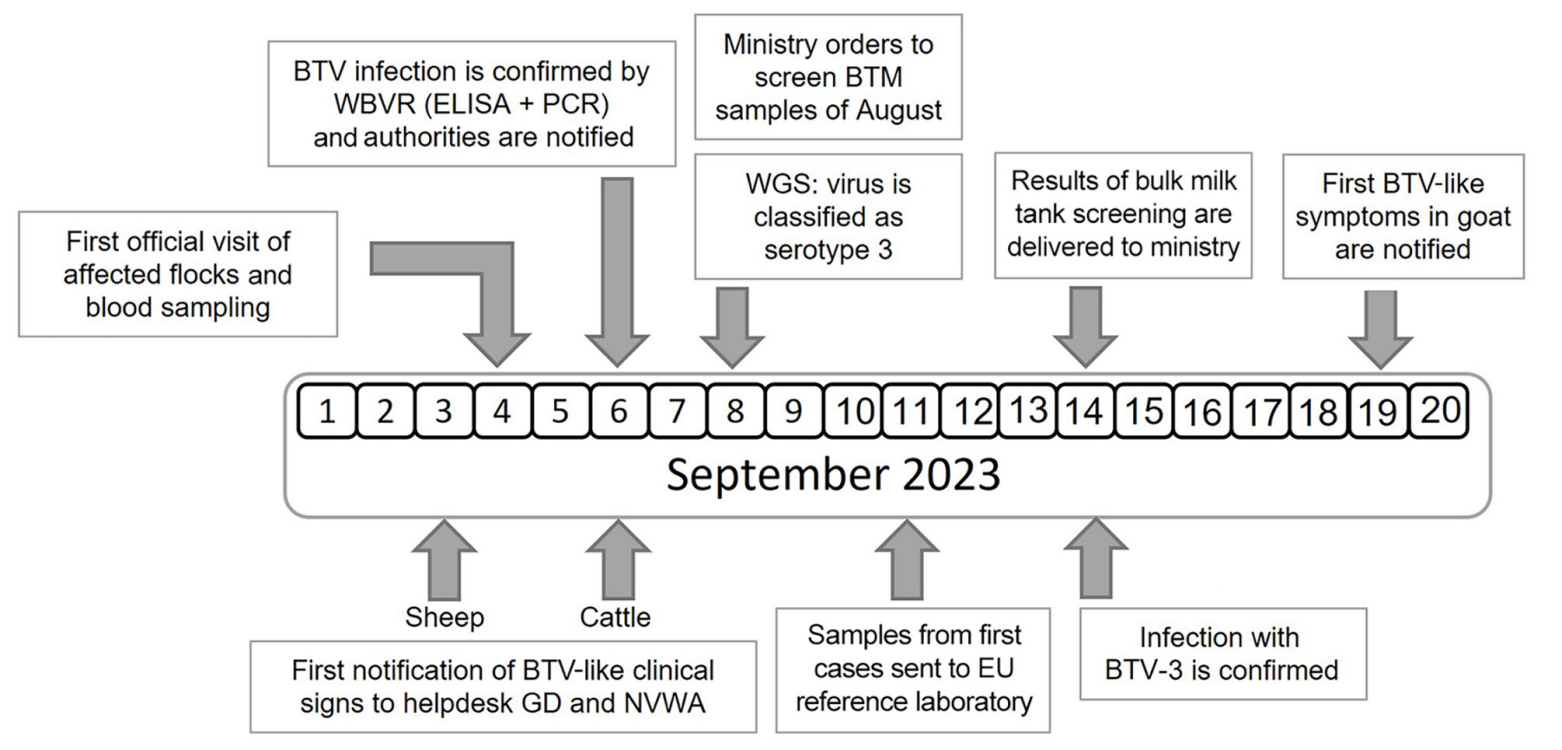
Timeline of the initial bluetongue outbreak caused by BTV-3 in the Netherlands in September 2023. BTV, bluetongue virus; BTV-3, BTV serotype 3; EU, European Union; GD, Gezondheidsdienst voor Dieren; NVWA, Netherlands Food and Consumer Product Safety Authority; WBVR, Wageningen Bioveterinary Research; WGS, whole-genome sequencing.

On September 6, we confirmed BTV infections by real-time RT-PCR and competitive ELISAs (Figure 1). Of the 7 blood samples taken from 5 sheep farms, 6 samples from 4 different farms had BTV-positive RT-PCR results; cycle threshold values were 23–31. BTV antibodies were found in blood samples from 5 of the 6 PCR-positive sheep; blocking was >90% in competitive ELISAs. We immediately reported the findings to the Ministry of Agriculture, Nature and Food Quality of the Netherlands, and requested new samples for confirmation and shipment to the EU Reference Laboratory for BTV, Center for Animal Health Research, National Center for Agricultural and Food Research and Technology, in Madrid, Spain. In addition, using nanopore technology, WBVR conducted whole-genome sequencing on the RT-PCR–positive samples. The first suspicion of BTV in cattle was also notified to the NVWA on September 6.

**Figure 2:**
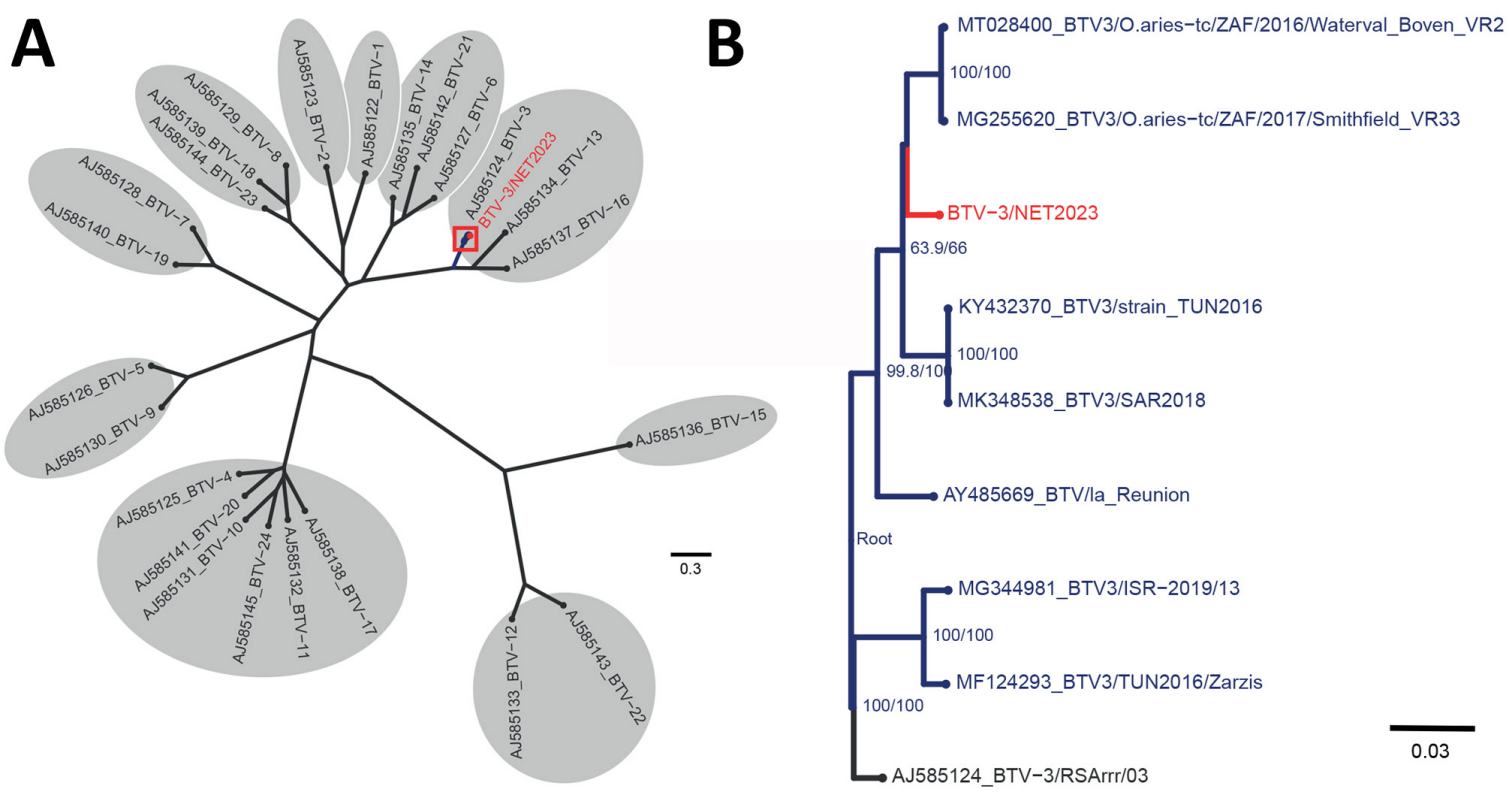
Phylogenetic analysis of BTV-3 variant found in livestock in the Netherlands, September 2023. Trees were obtained by using the maximum-likelihood method. A) Initial phylogenetic comparison of genome segment 2 sequence of the emerging BTV-3/NET2023 variant from the Netherlands with segment 2 sequences from notifiable BTV reference strain serotypes 1–24. B) Available and closely related genome segment 2 sequences from different BTV-3 strains selected for detailed phylogenetic analysis. Unrooted tree branches have bootstrap values indicated at the nodes. GenBank accession numbers are included in sequence names. Scale bars indicate nucleotide substitutions per site. BTV, bluetongue virus; BTV-3, BTV serotype 3.

On September 8, three blood samples from sheep sampled at 3 unrelated farms showed sufficient sequence coverage per nucleotide (range 30–2,570 reads) (Table 1) to reliably determine contig sequences for all 10 genome segments for those 3 samples. Contig sequences derived from individual samples were 100% identical. Contigs represented full-length sequences of Seg-1–Seg-9, including the 5′ and 3′ termini. Contigs of Seg-10 were incomplete and were completed by using Sanger sequencing, except for the ultimate 22 nt at the 3′ end, corresponding to the amplification primer. Phylogenetic analysis of Seg-2, which encodes the serotype-dominant VP2 protein, along with prototypic isolates of WOAH-notifiable BTV serotypes 1–24 identified the causative BT agent as BTV-3, which was designated as variant BTV-3/NET2023. Phylogenetic clustering of sequences was also observed with serotypes 13 and 16, confirming previous genetic analysis (Figure 2, panel A) (*22*). On the basis of the phylogenetic tree, WBVR announced that genotyping revealed the BT outbreak was caused by BTV-3 because of the high homology with known serotype 3 isolates. Detailed phylogenetic analysis showed a close relationship with Seg-2 from BTV-3 isolates from Italy and Tunisia (Figure 2, panel B). Phylogenetic analyses of other genome segments of BTV-3/NET2023 did not indicate a particular ancestor but had close identity (>97%) to genome segments of various other sequenced BTV isolates deposited in GenBank (Table 2).

**Table 1:** Average sequence coverage for each genome segment of 3 BTV-3 isolates in study of emergence of BTV-3 in the Netherlands, September 2023*

Sample no.	Ct value	Average no. reads/nucleotide for each genome segment									
		Seg-1	Seg-2	Seg-3	Seg-4	Seg-5	Seg-6	Seg-7	Seg-8	Seg-9	Seg-10
23014055	23.3	431	898	548	1,386	642	2,570	664	4,311	1,905	200
23014071	28.6	72	112	76	149	99	135	107	223	113	30
23014098	25.2	217	313	228	470	192	488	258	630	283	45

*Three blood samples from sheep were collected at 3 unrelated farms; isolated virus was subjected to whole-genome sequencing. All 10 RNA genome segments were sequenced. BTV-3, bluetongue virus serotype 3; Ct, cycle threshold; Seg, genome segment.

**Table 2:** Percentage homology between BTV-3/NET2023 variant and closest isolates deposited in GenBank in study of emergence of BTV-3 in the Netherlands, September 2023*

Segment	Virus protein	Highest % identity†	Isolate name	GenBank accession no.
Seg-1	VP1	97.69	BTV-8/2020_13	OQ860824.1
Seg-2	VP2	98.09	BTV-3/ZIM2002/01	AJ585179.1
Seg-3	VP3	98.30	BTV-5/O.aries-tc/ZAF/2011/Benoni_01012015	MG255451.1
Seg-4	VP4	98.37	BTV-3/TUN2016/Zarzis	MF124295.1
Seg-5	NS1	98.42	BTV-1/ISR-2050/19	OM502356.1
Seg-6	VP5	97.50	BTV-3/O.aries-tc/ZAF/2017/Smithfield_VR33	MG255623.1
Seg-7	VP7	98.27	BTV-3/O.aries-tc/ZAF/2016/Waterval_Boven_VR22	MT028405.1
Seg-8	NS2	97.69	BTV-2/O.aries-tc/ZAF/2017/Queenstown_VR18	MG255577.1
Seg-9	VP6/NS4	97.43	BTV-4/SPA2003/03	KP821911.1
Seg-10	NS3/NS3a	98.42	BTV-18/BT32/76	JX272448.1

*BTV-3/NET2023 variant was isolated from sheep in the Netherlands in September 2023 and genome segments were compared with those of BTV isolates deposited in GenBank. BTV-3, bluetongue virus serotype 3; NS, nonstructural protein; Seg, genome segment; VP, virus protein. †Highest % identity (>97%) compared with BTV-3/NET2023.

On September 11, WBVR confirmed BTV in newly collected serum and EDTA blood samples from all 4 initially infected sheep farms and sent the samples to the EU Reference Laboratory for confirmation and serotyping by serotype-specific real-time RT-PCR. On September 14, the EU Reference Laboratory confirmed the results by using the WOAH-recommended RT-PCR test targeting Seg-10. RT-PCR specific for serotypes 3, 4, and 8 clearly confirmed serotype 3, and the results were immediately forwarded to the Ministry of Agriculture, Nature and Food Quality of the Netherlands and NVWA.

On September 19, the first suspicion of BTV in a goat was notified to the NVWA; specimens collected from 1 goat were positive for BTV by real-time RT-PCR. In addition, BTV-3/NET2023 isolation from sheep EDTA blood from the initial 4 farms was successful by using *Culicoides*-derived KC cells (*23*).

### Clinical Manifestations in Sheep, Cattle, and Goats

**Figure 3:**
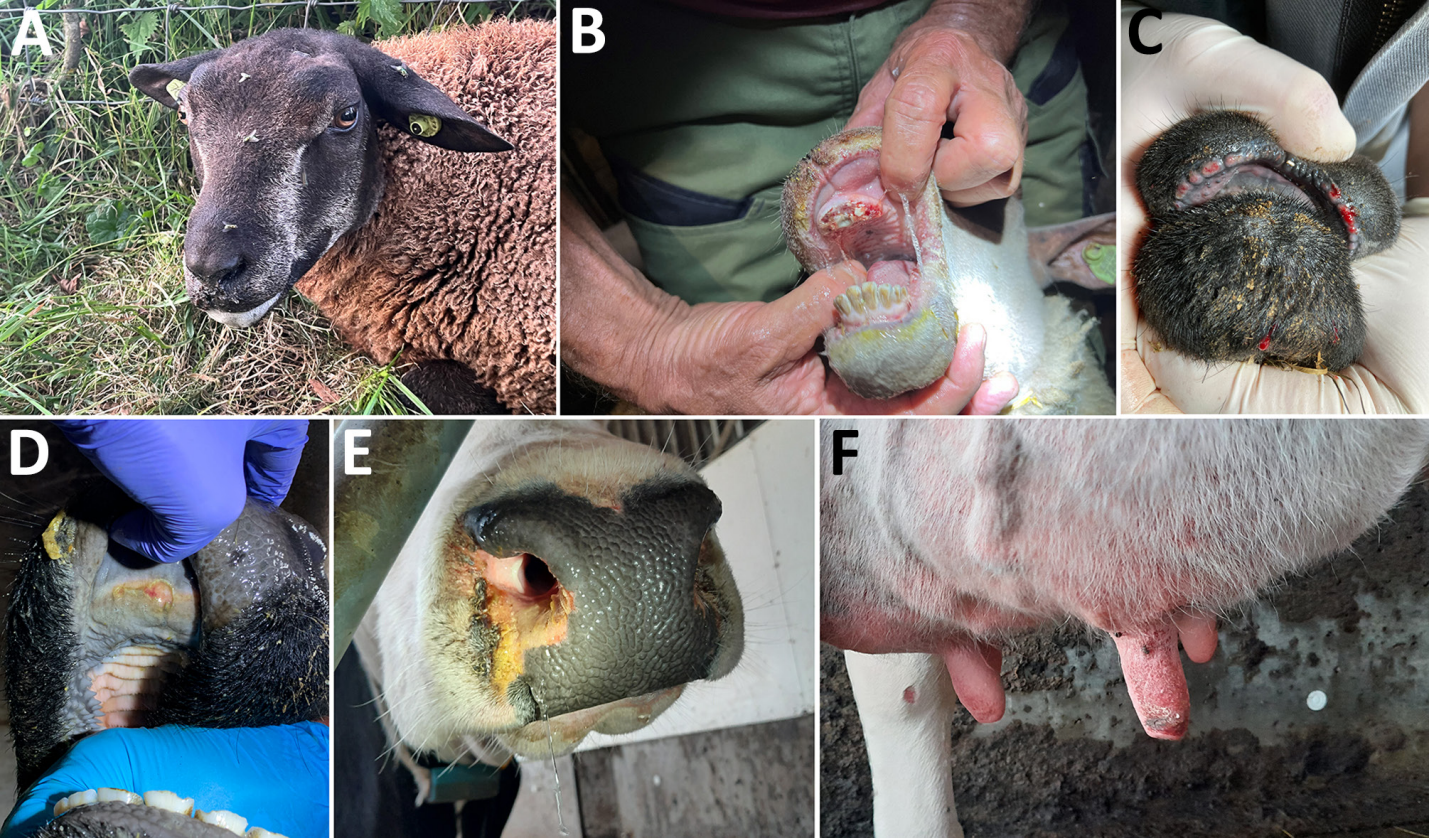
Clinical manifestations of bluetongue caused by bluetongue virus serotype 3 (BTV-3) variant infections in sheep and cattle in study of emergence of BTV-3 in the Netherlands, September 2023. A–C) Hypersalivation (A), erosion of the oral mucous membranes (B), and bleeding of the lips (C) were observed in sheep infected with the BTV-3 variant. D–F) Ulceration of the oral mucous membrane (D), crust formation at the nostrils (E), and necrosis of the teats (F) were detected in cattle infected with the BTV-3 variant.

BTV-3–infected sheep showed signs of fever, lethargy, hypersalivation, ulcerations and erosions of the oral and nasal mucous membranes, facial edema, lesions of the coronary band, lameness, and death (Figure 3). Several days after the initial outbreak confirmation in sheep, clinical signs were also reported in cattle. Clinical signs observed in cattle were fever, apathy, conjunctivitis, nasal discharge, erosions and crust formation on lips and nostrils, ulcerations and erosions of oral mucosa, edema of the nose, coronitis, and superficial necrosis of teats. The goat reported on September 19 showed signs of edema of the lips and fever (Appendix Figure 2).

**Figure 4:**
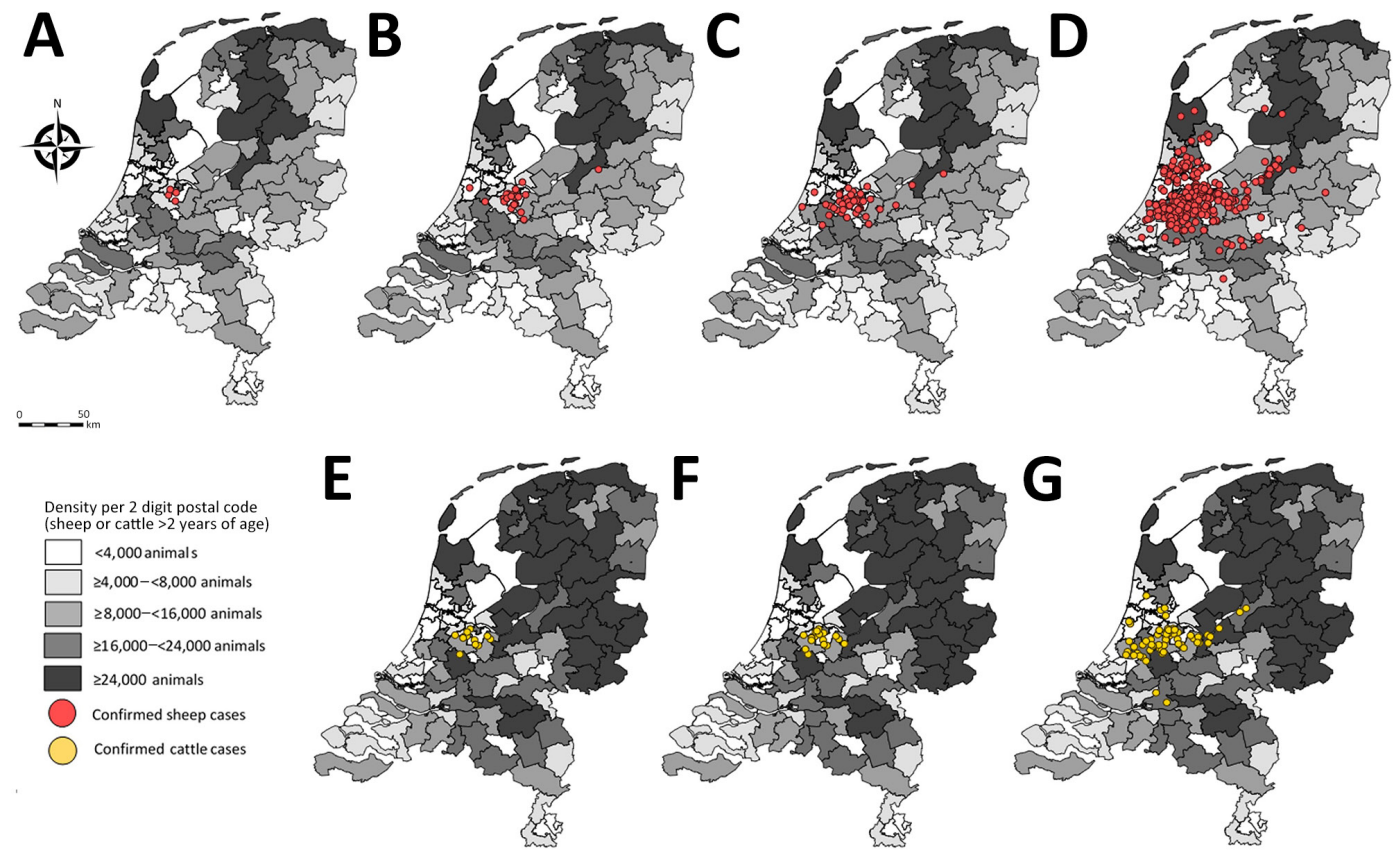
Density of sheep or cattle per 2-digit postal code and number of confirmed cases of bluetongue virus serotype (BTV-3)–positive flocks or herds in the Netherlands, September 2023. Gray shading indicates the density of animals. A–D) Distribution of sheep flocks infected with BTV-3. A) Initial 4 cases of BTV-3–infected sheep flocks notified on September 3, 2023. Confirmed sheep cases during calendar week 36 (B), calendar week 37 (C), and calendar week 38 (D). E–G) Distribution of cattle herds infected with BTV-3. Confirmed cases of infected cattle herds during calendar week 36 (E), calendar week 37 (F), and calendar week 38 (G).

After the outbreak began, the number of notifications increased rapidly for both sheep flocks and cattle herds (Figure 4). The initial cases included 4 sheep farms. One week later (calendar week 36), a total of 25 sheep flock and 12 cattle herd notifications were confirmed as BTV-3–positive by RT-PCR. In the second week (calendar week 37), the total number of positive suspicions increased to 18 sheep flocks and 55 cattle herds. In the third week (calendar week 38), the total number of PCR-positive BTV-3–diagnosed flocks or herds increased to 324 sheep flocks, 61 cattle herds, and 1 goat herd. 

### Retrospective Study

**Figure 5:**
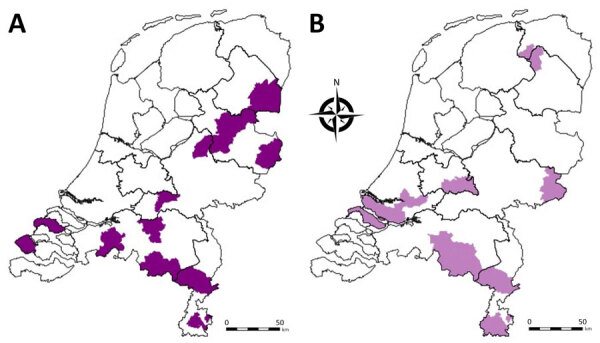
Geographic distribution of bulk milk samples in the Netherlands tested for bluetongue virus (BTV) antibodies in study of emergence of BTV serotype 3 in September 2023. Milk samples were collected in August 2023. Purple shading indicates locations of dairy cattle herds that had animals considered potential positive or positive for BTV-specific antibodies by using ELISA. A) Herds having evidence of vaccination in the previous 5 years. B) Herds having no evidence of vaccination in the previous 5 years.

To investigate whether the BT outbreak began at the 4 initially-infected sheep farms, we screened bulk tank milk samples submitted in August from cattle farms for routine BTV antibody testing. Of the 991 bulk tank milk samples, 955 tested negative, 8 tested potentially positive, and 28 tested positive for BTV antibodies; antibody prevalence was 2.8% (95% CI 1.9%–4.1%) (Table 3). However, 24 of 36 bulk tank milk samples showing potentially positive or positive results (67%) were from farms that had a proven history of animal vaccination against BTV-8. BTV antibodies that could not be linked to a recorded history of BTV vaccination were found in 12 of 991 bulk tank milk samples. However, the 12 antibody-positive herds were not clustered, and 7 of those 12 herds were located near the borders with Belgium and Germany, suggesting a high likelihood that those farms in the Netherlands might have vaccinated their animals because of the presence of BTV-8 in Belgium and Germany (Figure 5). Altogether, no area in the Netherlands had a high seroprevalence for BTV antibodies in August 2023 according to cow milk sampling, and no BTV-specific antibodies were found in the region where the initial BTV notifications were made. Therefore, the 4 BT index cases occurred in the 4 initially affected sheep farms.

**Table 3:** Herd-level BTV antibody results for 991 cow bulk milk samples collected in August 2023 in study of emergence of BTV-3, the Netherlands*

Compartment	No. dairy herds	No. herds tested	No. negative bulk milk samples	No. milk samples with potentially positive or positive result			Antibody-positive milk samples, % (95% CI)†
				Total	Evidence of BTV vaccination	No evidence of BTV vaccination	
1	1,669	49	49	0	0	0	0 (0–5.9)
2	678	49	49	0	0	0	0 (0–5.9)
3	880	51	51	0	0	0	0 (0–5.7)
4	1,040	49	48	1	0	1	2.0 (0.1–10.9)
5	963	47	45	2	2	0	0 (0–6.4)
6	352	49	49	0	0	0	0 (0–5.9)
7	562	52	52	0	0	0	0 (0–5.6)
8	1,332	50	49	1	1	0	0 (0–5.9)
9	610	51	51	0	0	0	0 (0–5.7)
10	546	48	47	1	0	1	2.1 (0.1–11.1)
11	1,412	48	46	2	1	1	2.1 (0.1–11.3)
12	942	50	49	1	0	1	2.0 (0.1–10.6)
13	464	53	52	1	1	0	0 (0–5.6)
14	162	48	44	4	2	2	4.3 (0.1–14.8)
15	96	50	50	0	0	0	0 (0–5.8)
16	457	47	46	1	1	0	0 (0–6.3)
17	293	52	43	9	7	2	4.4 (0.1–15.1)
18	778	52	47	5	5	0	0 (0–6.2)
19	361	48	43	5	3	2	4.4 (0.1–15.1)
20	145	48	45	3	1	2	4.3 (0.1–14.5)
Total	13,742	991	955	36	24	12	1.2 (0.1–2.2)

*Bulk milk samples were collected from dairy cattle herds in 20 compartments of the Netherlands in August 2023 and retrospectively analyzed to determine if BTV infections occurred before the detection of BTV-3 in sheep in September. BTV, bluetongue virus. †Determined for cows that had no evidence of vaccination against BTV.

## Discussion

We describe the actions taken after a novel BTV-3 strain emerged in sheep and cattle in the Netherlands. Initially, sheep from 4 farms showed clinical signs of fever, lethargy, hypersalivation, ulcerations, erosions of the oral and nasal mucous membranes, or sudden death. The sheep were positive for BTV by real-time RT-PCR, and all but 1 showed seroconversion by using a competition ELISA. Whole-genome sequencing using nanopore technology showed the full virus genome sequence could be characterized quickly, and the generated nucleotide sequence of Seg-2 aligned with other BTV-3 sequences. We investigated BTV epidemiology during the month before the first cases were reported in sheep by retrospectively testing bulk tank cow milk; however, either no high seroprevalence was observed or seropositive samples were found within the region where the initial cases were detected. A very low number of antibody-positive bulk milk samples (n = 12) were found that could not be linked to previous vaccination. It is possible that the cows from those herds were still vaccinated, but that possibility could not be substantiated on the basis of available data. In addition, those findings might have been false positives despite the high specificity of the ELISA (*21*). We concluded that a massive spread of BTV did not occur before the first detection of BTV-3 in the sheep farms, which agrees with the findings of a retrospective analysis of 1,003 sheep serum samples from 89 flocks that indicated a 3.4% BTV herd prevalence in August (I.M.G.A. Santman-Berends et al., unpub. data). As of March 12, 2024, animals from a total of 4,371 farms or holdings have been confirmed as BTV-3 positive by real-time PCR.

Early detection of diseases by clinical diagnosis remains challenging, especially for unpredicted nonendemic diseases. BT displays a wide and nonspecific spectrum of clinical manifestations in ruminants, such as fever, hypersalivation, lameness, edema, and sudden death. BT disease severity in sheep and cattle overlaps with several other endemic infections, such as orf, dermatophilosis, haemonchosis, pasteurellosis, strawberry footrot, and photosensitization, which are relevant, differentially diagnosed endemic conditions in sheep. Malignant catarrhal fever and photosensitization can cause signs similar to BT in cattle (*24*,*25*). Awareness of BTV-like symptoms by veterinarians is also of great importance for other notifiable diseases, such as foot-and-mouth disease, peste des petits ruminants, sheep and goat pox, and epizootic hemorrhagic disease, and should be notified to the official authorities when suspected.

During a BT outbreak, communication creates increased awareness among veterinarians and farmers, which might lead to an increase in false BT notifications because of nonspecific clinical signs. Therefore, education and training of veterinarians and livestock farmers about the clinical manifestations of BT and other diseases remains critical, especially for notifiable diseases that have not occurred for an extended time, because many veterinarians might not have seen the clinical symptoms in their practice. Only a laboratory diagnosis can and should rapidly differentiate between notifiable diseases to support a clinical diagnosis. Nevertheless, in the outbreak described in this study, the emerging BT disease was detected successfully at an early stage.

The rapid spread of BTV after the initial emergence shows that indigenous *Culicoides* spp. midges in the Netherlands are competent vectors for transmitting BTV-3/NET2023. The BTV vector, the *C*. *imicola* midge, found predominantly in Africa and Asia, is not found in northwestern Europe, and BTV-6 introduction in the Netherlands in 2008 showed that the outbreak dies out when indigenous *Culicoides* spp. midges are unable to effectively transmit the virus (*26*). BTV-3/NET2023 is the second BTV variant, after BTV-8/NET2006, that has been successfully transmitted by indigenous biting midge species in northwestern Europe (*27*). BTV-8/NET2006 is transmitted by indigenous biting midge species of the *C*. *obsoletus* complex, including *C. obsoletus*, *C.* *scoticus*, *C.* *dewulfi*, and *C.* *chiopterus* (*28*–*30*). Entomologic research is needed to identify the biting midge species involved in BTV-3/NET2023 transmission.

The geographic origin and route of introduction of BTV-3/NET2023 into the Netherlands are unknown. Phylogenetic analysis of Seg-2 shows clustering with other BTV-3 Seg-2 sequences, including geographically close BTV-3 variants. However, BTV-3 has only been described in Europe in Sicily and Sardinia, Italy, and the few sequences available from those virus isolates show a relatively high variation compared with BTV-3/NET2023 sequences from the Netherlands. In addition, other genome segment sequences did not show high homology with sequences from different BTV-3 variants. Therefore, tracing the origin of BTV-3/NET2023 has been difficult. The segmented genome of BTV enables reassortment, also known as antigenic shift, between variants, which further hampers unravelling the geographic source of BTV-3/NET2023. The virus was likely introduced into the Netherlands from a distant source because the neighboring countries Belgium and Germany have had BT-free status since June 2023. Although the source and route of BTV-3/NET2023 is unclear, yearly monitoring and the findings from this retrospective study indicate that virus circulation began in September 2023 within the Netherlands.

In conclusion, after a decade of having BT-free status, the Netherlands saw BTV-3 emerge in 2023, causing clinical signs and death in sheep and cattle. The causative virus strain is designated BTV-3/NET2023. The source, geographic origin, and introduction route of BTV-3/NET2023 are unknown, but virus circulation has rapidly expanded. BTV-3/NET2023 is transmitted by indigenous biting midges, but the vector-competent midge species has not yet been identified. Continuous monitoring and molecular diagnostic testing of sheep, cattle, and goats will be needed to determine virus spread, and new vaccination and other prevention strategies will be required to prevent BTV circulation within the Netherlands and Europe.

Appendix Additional information for emergence of bluetongue virus serotype 3, the Netherlands, September 2023.
