# Supplementary material for: Emergence of Bluetongue Virus Serotype 3, the Netherlands, September 2023
Source: Emerg Infect Dis. 2024 Aug;30(8):1552–61. doi: 10.3201/eid3008.231331 (PMC11286052; doi:10.3201/eid3008.231331)
Supplement: Appendix — Additional information for emergence of bluetongue virus serotype 3, the Netherlands, September 2023. [file 23-1331-Techapp-s1.pdf]

Article DOI: https://doi.org/10.3201/eid3008.231331

EID cannot ensure accessibility for supplementary materials supplied by authors. Readers who have difficulty accessing supplementary content should contact the authors for assistance.

Emergence of Bluetongue Virus Serotype 3, the Netherlands, September 2023

Appendix

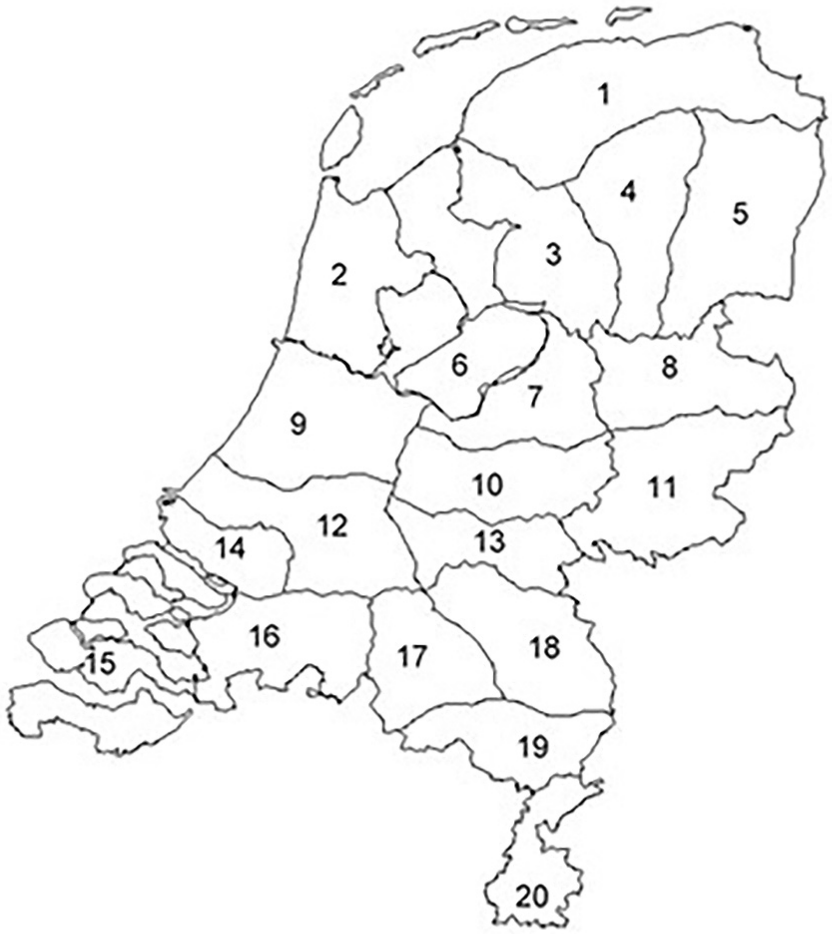

Appendix Figure 1. Location of each of the 20 compartments within the Netherlands proposed by the European Union Commission Decision 2005/393/EC.

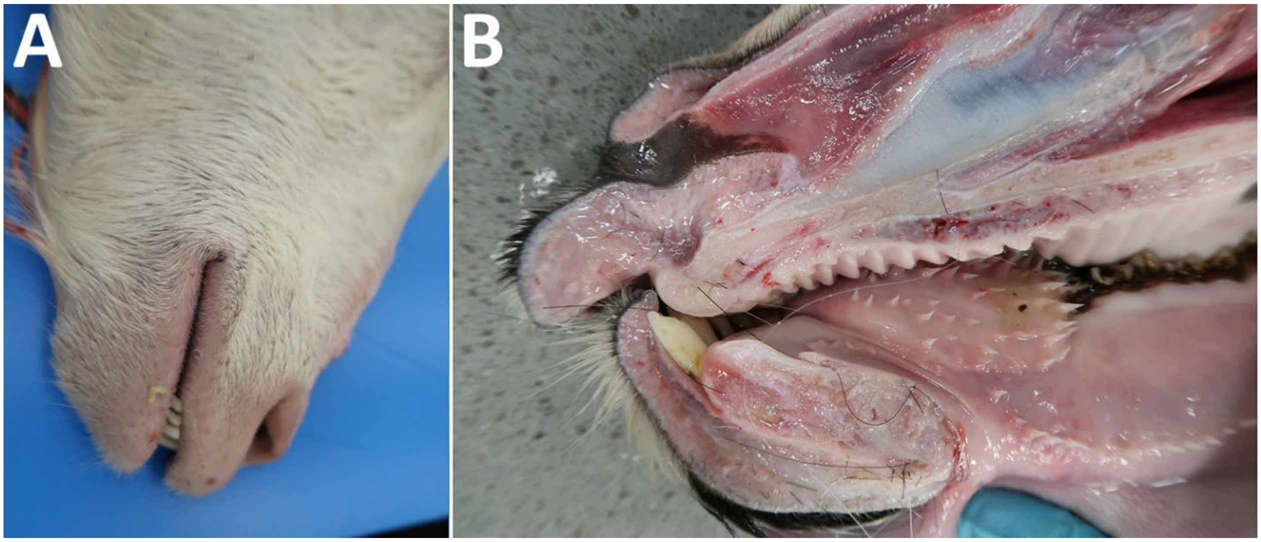

Appendix Figure 2. Edema formation in the head of a goat caused by bluetongue virus serotype 3 infection in the Netherlands. Bluetongue disease was reported in a goat on September 19, 2023, and showed signs of edema of the lips along with fever. A) Goat head. B) Cross-section of goat head.
